# Supplementary material for: A hybrid security system for drones based on ICMetric technology
Source: PLoS One. 2023 Mar 21;18(3):e0282567. doi: 10.1371/journal.pone.0282567 (PMC10030017; doi:10.1371/journal.pone.0282567)

PLOS ONE

A Hybrid Security System for Drones Based on ICMetric Technology

--Manuscript Draft--

**Manuscript Number:**	PONE-D-21-31928
**Article Type:**	Research Article
**Full Title:**	A Hybrid Security System for Drones Based on ICMetric Technology
**Short Title:**	A Hybrid Security System for Drones
**Corresponding Author:**	Khattab M. Ali Alheeti<br>University of Anbar<br>Ramadi, IRAQ
**Keywords:**	Drones, security, intrusion detection
**Abstract:**	Recently, the number of drones has increased, and the illegal and malicious use of drones has become prevalent. The dangerous and wasteful effects are substantial, and the probability of attacks is very high. Therefore, a detection and protection system is needed. This paper aims to design and implement an intelligent detection system for the security of unmanned aerial vehicles (UAVs)/drones. The proposed detection system is heavily based on utilizing ICMetric technology to exploit low-level device features for detection. This technology extracts the accelerometer and gyroscope sensors' bias to create a unique number known as the ICMetric number. Hence, ICMetric numbers represent additional features integrated into the dataset used to detect drones. In this study, the classification is performed using a deep neural network (DNN). The experimental results prove that the proposed system achieves high levels of detection and performance metrics.
**Order of Authors:**	Khattab M. Ali Alheeti
	Fawaz Khaled Alarfaj
	Mohammed Alreshoodi
	Naif Almusallam
	Duaa Al Dosary
**Additional Information:**	
**Question**	**Response**
**Financial Disclosure**<br><br>Enter a financial disclosure statement that describes the sources of funding for the work included in this submission. Review the submission guidelines for detailed requirements. View published research articles from *PLOS ONE* for specific examples.<br><br>This statement is required for submission and **will appear in the published article** if the submission is accepted. Please make sure it is accurate.	The authors extend their appreciation to the Deanship of Scientific Research at Imam Mohammad Ibn Saud Islamic University for funding this work through Research Group no. RG-21-51-01.

Powered by Editorial Manager® and ProduXion Manager® from Aries Systems Corporation

**Unfunded studies**<br>Enter: *The author(s) received no specific funding for this work.*<br><br>**Funded studies**<br>Enter a statement with the following details:<br>• Initials of the authors who received each award<br>• Grant numbers awarded to each author<br>• The full name of each funder<br>• URL of each funder website<br>• Did the sponsors or funders play any role in the study design, data collection and analysis, decision to publish, or preparation of the manuscript?<br>• **NO** - Include this sentence at the end of your statement: *The funders had no role in study design, data collection and analysis, decision to publish, or preparation of the manuscript.*<br>• **YES** - Specify the role(s) played.<br><br>* typeset	
**Competing Interests**<br><br>Use the instructions below to enter a competing interest statement for this submission. On behalf of all authors, disclose any competing interests that could be perceived to bias this work—acknowledging all financial support and any other relevant financial or non-financial competing interests.<br><br>This statement is required for submission and **will appear in the published article** if the submission is accepted. Please make sure it is accurate and that any funding sources listed in your Funding Information later in the submission form are also declared in your Financial Disclosure statement.<br><br>View published research articles from *PLOS ONE* for specific examples.	The authors have declared that no competing interests exist.

Powered by Editorial Manager® and ProduXion Manager® from Aries Systems Corporation

**NO authors have competing interests**<br><br>Enter: *The authors have declared that no competing interests exist.*<br><br>**Authors with competing interests**<br><br>Enter competing interest details beginning with this statement:<br><br>*I have read the journal's policy and the authors of this manuscript have the following competing interests: [insert competing interests here]*<br><br>* typeset	
**Ethics Statement**<br><br>Enter an ethics statement for this submission. This statement is required if the study involved:<br><br>• Human participants<br>• Human specimens or tissue<br>• Vertebrate animals or cephalopods<br>• Vertebrate embryos or tissues<br>• Field research<br><br>Write "N/A" if the submission does not require an ethics statement.<br><br>General guidance is provided below. Consult the submission guidelines for detailed instructions. **Make sure that all information entered here is included in the Methods section of the manuscript.**	N/A

Powered by Editorial Manager® and ProduXion Manager® from Aries Systems Corporation

**Format for specific study types**<br><br>**Human Subject Research (involving human participants and/or tissue)**<br>• Give the name of the institutional review board or ethics committee that approved the study<br>• Include the approval number and/or a statement indicating approval of this research<br>• Indicate the form of consent obtained (written/oral) or the reason that consent was not obtained (e.g. the data were analyzed anonymously)<br><br>**Animal Research (involving vertebrate animals, embryos or tissues)**<br>• Provide the name of the Institutional Animal Care and Use Committee (IACUC) or other relevant ethics board that reviewed the study protocol, and indicate whether they approved this research or granted a formal waiver of ethical approval<br>• Include an approval number if one was obtained<br>• If the study involved *non-human primates*, add *additional details* about animal welfare and steps taken to ameliorate suffering<br>• If anesthesia, euthanasia, or any kind of animal sacrifice is part of the study, include briefly which substances and/or methods were applied<br><br>**Field Research**<br><br>Include the following details if this study involves the collection of plant, animal, or other materials from a natural setting:<br>• Field permit number<br>• Name of the institution or relevant body that granted permission	
**Data Availability**<br><br>Authors are required to make all data underlying the findings described fully available, without restriction, and from the time of publication. PLOS allows rare exceptions to address legal and ethical concerns. See the PLOS Data Policy and FAQ for detailed information.	Yes - all data are fully available without restriction

Powered by Editorial Manager® and ProduXion Manager® from Aries Systems Corporation

A Data Availability Statement describing where the data can be found is required at submission. Your answers to this question constitute the Data Availability Statement and **will be published in the article**, if accepted.<br><br>**Important:** Stating 'data available on request from the author' is not sufficient. If your data are only available upon request, select 'No' for the first question and explain your exceptional situation in the text box.<br><br>Do the authors confirm that all data underlying the findings described in their manuscript are fully available without restriction?	
**Describe where the data may be found in full sentences. If you are copying our sample text, replace any instances of XXX with the appropriate details.**<br><br>• If the data are **held or will be held in a public repository**, include URLs, accession numbers or DOIs. If this information will only be available after acceptance, indicate this by ticking the box below. For example: *All XXX files are available from the XXX database (accession number(s) XXX, XXX.).*<br>• If the data are all contained **within the manuscript and/or Supporting Information files**, enter the following: *All relevant data are within the manuscript and its Supporting Information files.*<br>• If neither of these applies but you are able to provide **details of access elsewhere**, with or without limitations, please do so. For example:<br><br>*Data cannot be shared publicly because of [XXX]. Data are available from the XXX Institutional Data Access / Ethics Committee (contact via XXX) for researchers who meet the criteria for access to confidential data.*<br><br>*The data underlying the results presented in the study are available from (include the name of the third party*	In this study, the dataset represents the Wi-Fi traffic data records used. Two types of data exist within this set: the bidirectional flow mode that compromised 54 features and the unidirectional flow mode that compromised 18 features

Powered by Editorial Manager® and ProduXion Manager® from Aries Systems Corporation

and contact information or URL).<br>• This text is appropriate if the data are owned by a third party and authors do not have permission to share the data.<br><br>* typeset	
Additional data availability information:	

Powered by Editorial Manager® and ProduXion Manager® from Aries Systems Corporation

Manuscript Click here to access/download;Manuscript;Manuscript .pdf

A Hybrid Security System for Drones Based on ICMetric Technology

Khattab M Ali Alheeti[1,*], Fawaz Khaled Alarfaj[2], Mohammed Alreshoodi[3], Naif Almusallam[2] and Duaa Al_Dosary[1]

1 Department of Computer Networking Systems, College of Computer Sciences & Information Technology, University of Anbar, Ramadi, Iraq
2 Imam Muhammad Ibn Saud Islamic University (IMSIU), Riyadh, Saudi Arabia
3 Department of Natural Applied Science, College of Unizah Community , Qassim University , Unizah, KSA

* co.khattab.alheeti@uoanbar.edu.iq

Abstract

What kind of detection system you are addressing?

Recently, the number of drones has increased, and the illegal and malicious use of drones has become prevalent. The dangerous and wasteful effects are substantial, and the probability of attacks is very high. Therefore, a detection and protection system is needed. This paper aims to design and implement an intelligent detection system for the security of unmanned aerial vehicles (UAVs)/drones. The proposed detection system is heavily based on utilizing ICMetric technology to exploit low-level device features for detection. This technology extracts the accelerometer and gyroscope sensors' bias to create a unique number known as the ICMetric number. Hence, ICMetric numbers represent additional features integrated into the dataset used to detect drones. In this study, the classification is performed using a deep neural network (DNN). The experimental results prove that the proposed system achieves high levels of detection and performance metrics.

Format: Please justify your text

Introduction

Unmanned aerial vehicles (UAVs), commonly called drones, have become more readily available, more common, and more sophisticated. UAVs support new capabilities, such as autonomous behaviour and increased data collection. Currently, due to their ease of use and accessibility, they are exploited for commercial purposes in various fields, such as surveying, delivering medicines and goods, public surveillance, agriculture, cartography, and first aid. The primary architecture of Detection System for UAVs is presented in Fig 1

Fig 1. Architecture of Detection System for UAVs [1].

why so sudden you introduce figure about detection while u are talking about the usage

The rapid and comprehensive development of UAVs has attracted the attention of researchers and prompted them to propose further research in the domains of defence/protection against attacks. Cybersecurity attacks can involve hacking, hijacking, or loss of the communication signals of the drone. It is also challenging to ensure the safety of UAVs moving in unwanted areas, which produces weak robustness against various cybersecurity attacks [1]. Due to the UAV network connection, it is easy

now you are talking about monitoring. Are u looking into monitoring or detecting?

for adversaries to target or control. Hence, an intelligent monitoring system for drones is required to ensure their safety and provide identification. In this paper, the identification and security of drones were achieved and ensured. The proposed approach is based on a new technology called an integrated circuit metric (ICMetric), which heavily depends on each device's internal behaviour.

i cant really picture how the ICMetric can be used in detection. And what the icmmetric look like

Using the ICMetric, low-level device features can be used to identify the device [2]. ICMetrics is a new technique based on the concept of feature extraction for digital devices. However, it depends on devices' special features to generate a single ICMetric number because every device is singular in its internal environment [3]. This ICMetric number is unique and may be used to achieve detection/identification of the device. Microelectromechanical system (MEMS) sensors are utilized to generate ICMetric numbers. In other words, the bias readings extracted from these sensors will create this unique security number. The main contributions of this paper are summarized below:

1. ICMetric technology is employed for the first time for drone security.
2. The use of ICMetric technology aims to generate single/ unique numbers by considering additional features for the detection process.
3. A deep neural network is used as a classifier during the training phase for the detection system

The rest of this paper is organized as follows: Section 2 presents related works. Section 3 presents the details of integrated circuit metric technology. Section 4 presents the methodology of the proposed system. Section 5 evaluates the efficiency of this approach through experimental results. Section 6 presents the discussions, and finally, Section 7 presents the conclusions and possibilities for future research.

Related Works

what are those various techniques

what are those exploitation?

who present the review?

what survey?

This section summarises the latest research in related fields. Gritzalis et al. presented a literature review of various sensor techniques proposed for preventing, detecting, identifying, and mitigating damage from rogue drones, as well as a detailed survey of drone incidents near airports [4]. In the malicious purposes field, drones are exploited for different uses, which were reviewed in [5], along with other detection methods. The aforementioned study also analyzed the vulnerabilities of drones exploited through communication links and smart devices and hardware. Additionally, a detailed review of drone usage for various purposes in areas such as civilian, terrorist, and military domains was presented. [6] classified intrusion detection system (IDS) mechanisms used with attacks and vulnerabilities in networked drone environments. The existing IDS mechanism classification is based on information gathering sources, detection methods, deployment strategies, IDS acknowledgement, detection states, and intrusion types. A survey aimed to analyze the security threats against unmanned aircraft systems [7]. This work summarized previous studies related to the security of unmanned aircraft systems.

here is related work or your methodology?

What result?

Meanwhile, [8] proposed a protection system based on a convolutional neural network (CNN) for drone detection. The system was divided into two stages, where the first aimed to detect moving objects, while the ensuing second stage intended to classify the object detected as a bird, drone, or background. The results proved that the proposed method could achieve accuracy comparable to other proposed methods. In [9], deep learning techniques were utilized to propose an object detection system for drone detection. Various CNNs were used in the detection system to evaluate and test the dataset. The results showed that the proposed method based on one of the CNNs presented better performance than others with respect to the training dataset. Other

research based on CNNs for presenting an algorithm to solve the problem of drone detection was proposed in [10]. The proposed algorithm yielded high recall and precision values.

In [11], the authors presented a new system for detecting and tracking UAVs through a single camera mounted on various UAVs. This approach can detect and track small UAVs efficiently with limited computing resources. For drone detection, a lighter, inexpensive, safer, and miniaturized radar system was proposed in [11]. The practical use of the system was proven by the results. Different types of anomalies were accurately detected by the intrusion detection system proposed for drone communication networks in [12]. An adaptive intrusion detection system for drone identification based on deep learning techniques was presented in [1]. In this work, to maintain the high true positive rate of the IDS, a support vector machine (SVM) was used. The results showed that the proposed IDS can be deployed against cybersecurity attacks on UAVs with high levels of accuracy, specificity, and sensitivity.

when u talk about proposed model are u referring yours?

in related work u are talking more on others

The increased frequency of attacks against drones requires an intelligent intrusion detection system for identification and protection. In [13], a novel model for intrusion detection in drones based on one-class classifiers was proposed. The proposed model was effective across multiple UAV platforms with platform-specific F1 scores of 99.56% and 99.73% for benign and malicious sensor readings. In [14], a framework for identifying malicious attacks on the sensors of small, unmanned aircraft systems was presented. To illustrate the proposed model, a spoofing attack on the global positioning system (GPS) was detected. Different conclusions were drawn using this model with a simulation dataset. [15] presented a framework for attack detection that could be used to protect the environment of medical delivery drones with active capability, delivery reliability, and other medical applications.

Having analyzed systems proposed in previous studies, this study can be distinguished from others in that it presents an identification/detection system based on the ICMetric technology that depends on individual devices' internal behavior to provide a unique number for identification. The proposed approach has been developed to train the proposed security system by using the deep neural network technique.

Integrated Circuit Metric Technology (ICMetric)

Encryption systems depend on calculations that rely upon the utilization of the secret key. In attempting to improve success, however, expanding the size of the key generally does not ensure the security of the framework or prevent theft. The ID of the device, dependent on the exceptional features of every device, serves to prevent theft. Other equipment procedures vary with respect to the ICMetric innovation in the choice of device attributes. Conventional fingerprinting strategies rely on qualities that are effortlessly designed to detect, misdirect or reduce the damage inflicted by aggressors. ICMetrics use a portion of the properties that expand the intricacy of ICMetric and are difficult for an attacker to predict, such as media access control (MAC) locations and serial numbers. Different features with uncommon application use are employed, such as perusing narratives, camera goals, normal client documents, and framework profiles [16].

what data is being used to generate the icMetric?

ICMetrics focuses on another strategy that can utilize the features from the equipment and programming climate of a framework. Each device is unique in its internal climate; thus, the features that make each device different can be utilized to generate a single, one-of-a-kind number for every device. This depends on the following ideas [3].

1. The ICMetric number is not stored in the framework and can be reproduced when required.

u are not explained in detail regarding ICMetric, how the device generate it and based on what. give some example

2. If the system is assaulted, there will be no burglary since the ICMetric is not stored.

3. The ICMetric number and any procedure results that depend on the ICMetric number will be changed if any change to the product, equipment or climate is enacted.

4. There is no need for storage in any format that can serve the function of device approval.

too brief of explanation of the phases iinvolved

ICMetric is a technology that has advanced alongside the security requirements for digital devices. ICMetrics exploit characteristics of the system to present identification. This represents a new technology that can extract software and hardware attributes. Each device is unique in its internal environment. Therefore, the features that make each device different from another can create an impressive number of known ICMetric numbers. These numbers are not saved and can be regenerated when required. As these numbers are not saved/stored, there will be no theft if the system is exposed to attacks [3]. The two phases of ICMetric are [17]:

Calibration Phase: This involves measurement of the desired feature values; generation of feature distributions for each feature illustrating the frequency of each occurrence of each discrete value for each sample device; and normalization of the feature distributions and, generating normalization maps for each feature.

Operation Phase: This involves generating the ICMetric number by exploiting the device's internal behaviour; this number is uniquely used as an additional feature for detection.

As discussed above, the generation of an ICMetric system requires two phases, which are presented in Fig 2.

Fig 2. Flow Diagrams of ICMetric Phases [18].

Methodology

In this paper, a detection system based on ICMetric technology is proposed to achieve drone security—some details of the proposed ICMetric detection system are presented in the following sections.

Deep Neural Network

Deep neural network (DNN) technology is the subfield of machine learning used in this study as a detection classifier. More specifically, a DNN is a classification learning algorithm that uses a machine learning concept to maximize the accuracy of prediction. Moreover, DNNs are practical, robust, and accurate and are becoming increasingly important when operating in the training and testing phases.

Here, a DNN is employed to train the dataset for the proposed detection system. The DNN has three main layers: an input layer that consists of 18 neurons, three hidden layers, and an output layer. This proposal relies on increasing the detection system accuracy and decreasing the false alarm rate. Each hidden layer consists of four neurons in each hidden layer, while the output layer features two that are normal and abnormal. The testing phase was utilized to determine the system's efficiency with respect to error rate, detection accuracy rate, and four alarm types (confusion matrix).

The measurements for choosing the best design of the DNN used in the proposed model relied on trial-and-error and the best ratio principle for neural network training.

In this study, the reason for designing a DNN with 18 neurons in the input layer relates to the number of features used and will be explained further in a later section. Moreover, according to the trial-and-error principle, the optimal number of neurons for designing the hidden layer is four layers with five neurons. The best DNN structure used is presented in Fig 3.

Fig 3. DNN Basic Structure.

The initial parameters that have a direct impact on the classification performance of the training phase used in the DNN are presented below Table 1 .

Table 1. Initial parameters.

Parameter	Value
Train Parameter learn	$1 * 10^{-7}$
Train Parameter goal	0
Gaussian Radial Basis Function	1
Train Parameter epochs	23
Train Parameter min_grad	$1 * 10^{-13}$
BoxConstraint	1^{e6}

,

Dataset Description

TThe consumer UAV market has developed substantially over recent years. Regardless of its enormous potential for monetary development through supporting different applications, the advancement of shopper UAVs presents expected dangers to public security and individual protection. To limit these dangers, it is critically necessary to proficiently distinguish and recognize attacking UAVs. Given that purchaser UAVs are normally utilized in a regular civilian environment, existing identification techniques (such as radar, vision, and sound) may become ineffectual in numerous situations. To avoid these issues, the encrypted Wi-Fi traffic information records of UAVs can be an exceptionally encouraging source to identify UAV intruders.

Notwithstanding precision, the expected runtime of the indicator technique is exceptionally significant considering that UAV gatecrashers fly many metres each second and acquire the forecast as quickly as expected. In this way, the precision and runtime of the forecast should be improved. Subsequently, pruning and working on the model would be exceptionally significant: for example, pruning the redundant highlight ages and blending the common calculations.

In this study, the dataset represents the Wi-Fi traffic data records used. Two types of data exist within this set: the bidirectional flow mode and its 54 features and the unidirectional flow mode with 18 features [19]. For this dataset, three types of drones have been utilized:

- Parrot Bebop 1
- DBPower UDI
- DJI Spark

Which are as shown in Fig 4:

Fig 4. Types of Drones Used in the Dataset [19].

In this paper, the unidirectional flow mode was used to test/ evaluate the security system's performance. Meanwhile, this dataset is composed of nine features that are explained in Table 2.

Table 2. Statistical Measures Features [19]

Feature ID: Name	Description
V_1 : mean	$\bar{x} = \frac{1}{N}\sum_{i=1}^{N} x(i)$
V_2 : median	the higher half value of a data sample
V_3 : MAD^1	MAD = $median(\lvert x(i) - median(x)\rvert)$
V_4 : STD^1	$\sigma = \sqrt{\frac{1}{N-1}\sum_{i=1}^{N}(x(i) - mean(x))^2}$
V_5 : Skewness	$\gamma = \frac{1}{N}\sum_{i=1}^{N}(x(i) - mean(x)/\sigma)^3$
V_6 : Kurtosis	$\beta = \frac{1}{N}\sum_{i=1}^{N}(x(i) - mean(x)/\sigma)^4$
V_7 : MAX	$H = (Max(x(i))\rvert_{i=1...N})$
V_8 : MIN	$L = (Min(x(i))\rvert_{i=1...N})$
V_9 : Mean Square (ms)	$MS = \frac{1}{N}\sum_{i=1}^{N}(x(i))^2$

,

The dataset is a combination of various UAVs and traffic behaviour modes.

Fig 5. Snapshot of dataset sample

ICMetric Number Generation

ICMetric generation starts once a device's features have been extracted. Singular features of devices were collected, and then statistical processes were provided. The creation of ICMetric required two phases: a calibration phase and an operational phase. These phases were applied as and when required, after which the ICMetric and any relevant information were disposed of. The ICMetric and related component information is rarely imparted during any period of creation or use. This system uses ICMetric technology to generate a unique ICMetric number used for identification/detection, which will be integrated into the dataset used in this research as additional features for identification. The generation of this number is based on a mathematical model and requires some statistical analysis for this purpose. The generation process requires a probability mass (??) function to determine the precise value from the bias reading because these readings are discrete random variables.

$$p(x) = \frac{1}{\sigma\sqrt{2n}} e^{\frac{-(x-\mu)^2}{2\sigma^2}}, \quad (1)$$

If $\bar{x}$ represents the mean, x represents a specific number of readings extracted from the accelerometer and gyroscope, while n represents a total number of readings [20].

$$\bar{x} = \frac{1}{n}\sum_{i=1}^{n} x_i, \quad (2)$$

For ICMetric generation, the standard deviation (σ^2) is calculated:

$$\sigma^2 = \sum_{i=1}^{n} P(x_i)(x_i - \bar{X})^2, \quad (3)$$

Furthermore, variance (s^2) is calculated:

$$s^2 = \frac{1}{n-1}\sum_{i=1}^{n}(x_i - \bar{X})^2, \quad (4)$$

The skewness distribution (S) is calculated by Eq (5) :

what is the data?

$$S = \frac{3(\bar{X} - m)}{s^2}, \quad (5)$$

the confidence interval was used as calculated by Eq (6) [2].

$$CI = \bar{X} \pm v\frac{\sigma}{\sqrt{n}}, \quad (6)$$

In this paper, a security system is proposed for detection in a drone's environment. The scheme is heavily based on readings generated by MEMS (gyroscope and accelerometer) sensors. However, the bias readings extracted from these sensors were utilized to create ICMetric numbers. An ICMetric number is a unique number that fulfilled a vital role as additional features were added to the dataset used in this work.

The Proposed ICMetric Detection System

The ICMetric technique depends heavily on measurable features, which were extracted from the properties of a particular digital device. The focus was on utilizing MEMS sensors in the form of gyroscopes and accelerometers.

For this research, a drone is required, where MEMS sensors are embedded and used to provide bias readings. These bias readings are employed to create an ICMetric number that is used as identification for the drone. The steps of the ICMetric detection system are shown in Fig 6.

Fig 6. ICMetric System Block Diagram

After dataset selection, the ICMetric number was generated by a simple mathematical model as mentioned. To generate an ICMetric number, two types of MEMS sensors are utilized: the accelerometer and gyroscope. Furthermore, bias readings were extracted from these sensors to generate ICMetric numbers used as additional features integrated into the drone's detection dataset. Two phases, the training and testing phases, were utilized to evaluate the proposed system's efficiency. Hence, DNN is being used as an intelligent classifier for system evaluation.

Experimental Results

After dataset selection, the system is ready for training/ testing phases to evaluate the performance of the proposed detection system—the total accuracy of the training reached 99.99%.

In this study, DNN is employed as an intelligent classifier to achieve more accurate results. The DNN used in this work consists of three layers: an input layer, hidden layers, and an output layer. The trial and error principle is utilised to select the number of hidden layers based on the accuracy rate. Table 3 shows the number of hidden layers that can affect the proposed detection system's performance metrics.

Table 3. Accuracy Rate with Different Numbers of Layers and Neurons

Number of Hidden Layers	Performance	Epochs	Time	Training Accuracy
Two hidden layers with 5 neurons	0.41	22	18_s	46.79%
Three hidden layers with 5 neurons	0.8	25	26_s	52.6%
Four hidden layers with five neurons	2.0099e16	38	29_s	100%
Six hidden layers with 5 neurons	0.373	11	31_s	99.96%

,

Fig 7. Training Phase with Optimal Number of Hidden Layers

According to Table 3, it is readily noticeable that the optimal number of hidden layers is four, with five neurons in each layer. Fig 7 shows the training phase with an optimal number of hidden layers with neurons.

Fig 8, Fig 9, and Fig 10 show the performance and training status of the DNN in this detection approach.

Fig 8. Training Performance of DNN - MSE

Fig 9. Training State of DNN - Gradient

Fig 10. Training Status of DNN - Validation

Some metrics were utilized to calculate accuracy, effectivity, confusion matrix, and recall. These were then calculated to evaluate the efficiency of the proposed system as presented below [21]:

$$Accuracy = \frac{Number\ of\ correctly\ classified\ patterns}{Total\ number\ of\ patterns}, \quad (7)$$

To measure and evaluate the proposed system performance, four types of alarms are needed: true positive (TP), true negative (TN), false positive (FP), and false negative (FN). These metrics are calculated depending on the equations in 8, 9, 10, and 11:

$$TP_{Rate} = \frac{TP}{TP + FN}, \quad (8)$$

$$TN_{Rate} = \frac{TN}{TN + FP}, \quad (9)$$

$$FN_{Rate} = \frac{FN}{FN + TP}, \quad (10)$$

$$FP_{Rate} = \frac{FP}{FP + TN}, \quad (11)$$

To evaluate efficiency, the proposed system tested for some metrics with a testing rate of 99%. Table 4 shows the classification system performance and the number of feature records used in the detection system that utilized ICMetric technology.

Table 4. Classification Accuracy Rate with ICMetric

**Round 1 with ICMetrics**					
**Class**	**Actual Dataset**	**Detection System**	**Match Record**	**Miss Type**	**Accuracy Ratio**
Normal	2805	2803	2803	0	99.92%
Abnormal	2195	2195	2195	0	100%
Unknown	0	2	0	2	Nan

**Round 2 with ICMetrics**					
**Class**	**Actual Dataset**	**Detection System**	**Match Record**	**Miss Type**	**Accuracy Ratio**
Normal	2805	2850	2850	0	100%
Abnormal	2150	2149	2149	0	99.95%
Unknown	0	1	0	1	Nan

**Round 3 with ICMetrics**					
**Class**	**Actual Dataset**	**Detection System**	**Match Record**	**Miss Type**	**Accuracy Ratio**
Normal	2773	2773	2773	0	100%
Abnormal	2227	2225	2225	0	99.91%
Unknown	0	2	0	2	Nan

,

According to Table 4, higher rates of accuracy were achieved. Moreover, a higher rate of accuracy proved the result of using a balanced dataset achieved by a trusted source in [19]. In addition, ICMetric numbers are generated by a simple mathematical model and integrated with the dataset, resulting in a high accuracy rate. Using the equations in 8, 9, 10, and 11, the confusion matrix or alarm rate was calculated. The results are shown in Table 5.

Table 5. Average Alarms Rate

**Alarms**	**Ratio**
True Positive	98.76%
True Negative	100%
False Negative	1.24%
False Positive	0%

,

The ratio of the correctly positively labelled results to the total number of all positive labels is known as the detection precision.

With various numbers of drones, the classification rate was calculated as presented in Table 6.

Table 6. Classification Rate with Various Numbers of Drones

**Number of Drones**	**Accuracy Ratio**	**Time**
4	94%	0.83s
5	91.82%	0.84s
6	90.27%	2.35s
7	87.2%	3.82s
8	83.61%	4.69s
9	82.7%	4.83s

,

To evaluate the DNN classifier's performance, the receiver operating characteristic

(ROC) curve was measured. ROC shows the performance of the metrics for classification problems in defining the threshold of a system. The true positive rate and the false positive rate are two parameters improved by plotting the ROC curve, as presented in Fig 11 and Fig 12.

Fig 11. ROC curve for DNN - FPR

Fig 11 shows the FP rate's plotted value as the decision threshold is varied to present the ROC curve.

Fig 12. ROC curve for DNN - TPR

Fig 12 shows the TP rate's plotted value as the decision threshold is varied to present the ROC curve.

Discussion

As presented in Tables 4, 5, and 6, the detection system that is heavily based on ICMetric technology and DNN as classifiers is more effective, fast and efficient, with low error rates in detecting abnormal drones. The proposed method has presented acceptable performance results for detecting malicious drones. In Table 6, we can notice that the proposed approach has greater malicious detection ability when the number of drones is increased. The proposed ICMetric system is compared with another ordinary system to prove that the proposed system can achieve better accuracy when drones increase, as shown in Table 7. However, we can easily notice that the proposed system's accuracy rate fluctuates because it is affected by many external factors, such as behavior, signal jamming, traffic mode, and mission type.

format: make sure the table not more than the writing area

Table 7. Comparison with Existing IDS systems

Name	Year	Accuracy	Dataset	Classifier	Security Target
[22]	2019	91.50%	KDD Cup 99	Particle Swarm Optimization (PSO)	Provide a solution to solve intrusion detection problem of UAV networks
[8]	2020	99.83%	Drone-vs-Bird challenge dataset	CNN	Propose a real-time drone detection algorithm to solve the task of drone detection
[23]	2016	81.00%	UAVs, contain specific sensors, to collect information	based on a Belief approach	Protect the UAVs against the most dangerous threats
[24]	2018	94.00%	UAV stores	SVM	detect malicious anomalies that threaten the network
[25]	2019	92.00%	GPS signals	artificial neural network	detect GPS spoofing signals

,

Fig 13 compares the proposed ICMetric system with another ordinary system (without ICMetric) to prove that the proposed system was more accurate when the number of drones increased. However, we can easily notice that the accuracy rate fluctuated because it was affected by many external factors, such as behaviour, signal jamming, traffic mode, and mission type. Finally, to demonstrate the performance of this proposed system, its performance was compared with existing IDS system UAVs. These systems exploit different specifications, models and learning algorithms.

In Table 7, the accuracy is utilized as a performance metric to enable comparison of the performance of the proposed system with those of different existing IDS methods.

Fig 13. Comparison between the Proposed System and Other IDS Systems

Consequently, it can be observed that our solution achieved a better accuracy rate of 99.99% compared to other IDS models for UAVs.

Conclusions and Future Directions

In this paper, an intelligent detection system was proposed for drone security against attacks depending on ICMetric technology. The proposed approach was implemented in the following phases: simulation, data collection, feature extraction, preprocessing, training, and final testing. The deep neural network technique process plays a significant role in improving the proposed system's accuracy rate. The results of the simulation showed that the proposed detection system is accurate with respect to correctly classifying normal and abnormal behaviours and can detect malicious states and isolate them. The accuracy rates for the training and testing phases were 99.99% and 99%, respectively.

In future works, the proposed system can be designed with other artificial intelligence techniques, such as support vector machines and linear discrimination analysis. In addition, the ICMetric can be extracted from ultrasound sensors equipped with a UAV.

Acknowledgement

The authors extend their appreciation to the Deanship of Scientific Research at Imam Mohammad Ibn Saud Islamic University for funding this work through Research Group no. RG-21-51-01.

References

1. Arthur MP. Detecting Signal Spoofing and Jamming Attacks in UAV Networks using a Lightweight IDS. In: 2019 International Conference on Computer, Information and Telecommunication Systems (CITS); 2019. p. 1–5.

2. Tahir H, Howells G, Hu H, Gu D, Mcdonald-Maier K. On the Incorporation of Secure Filter in ICMetrics Group Communications. In: 2014 Fifth International Conference on Emerging Security Technologies; 2014. p. 77–81.

3. Tahir S, Afzal M, Tahir M. An ICMetric based key generation scheme for controlled group communication. In: IISA 2014, The 5th International Conference on Information, Intelligence, Systems and Applications; 2014. p. 373–378.

4. Lykou G, Moustakas D, Gritzalis D. Defending Airports from UAS: A Survey on Cyber-Attacks and Counter-Drone Sensing Technologies. Sensors. 2020;20(12). doi:10.3390/s20123537.

5. Yaacoub JP, Noura H, Salman O, Chehab A. Security analysis of drones systems: Attacks, limitations, and recommendations. Internet of Things. 2020;11:100218. doi:https://doi.org/10.1016/j.iot.2020.100218.

6. Choudhary G, Sharma V, You I, Yim K, Chen IR, Cho JH. Intrusion Detection Systems for Networked Unmanned Aerial Vehicles: A Survey. In: 2018 14th International Wireless Communications Mobile Computing Conference (IWCMC); 2018. p. 560–565.

7. Li L, Qu K, Lin KY. A Survey on Attack Resilient of UAV Motion Planning*. In: 2020 IEEE 16th International Conference on Control Automation (ICCA); 2020. p. 558–563.

8. Seidaliyeva U, Akhmetov D, Ilipbayeva L, Matson ET. Real-Time and Accurate Drone Detection in a Video with a Static Background. Sensors. 2020;20(14):3856. doi:10.3390/s20143856.

9. Saqib M, Daud Khan S, Sharma N, Blumenstein M. A study on detecting drones using deep convolutional neural networks. In: 2017 14th IEEE International Conference on Advanced Video and Signal Based Surveillance (AVSS); 2017. p. 1–5.

10. Aker C, Kalkan S. Using Deep Networks for Drone Detection; 2017.

11. Park S, Kim Y, Lee K, Smith AH, Dietz JE, Matson ET. Accessible Real-Time Surveillance Radar System for Object Detection. Sensors. 2020;20(8):2215. doi:10.3390/s20082215.

12. Condomines JP, Zhang R, Larrieu N. Network intrusion detection system for UAV ad-hoc communication: From methodology design to real test validation. Ad Hoc Networks. 2019;90:101759. doi:https://doi.org/10.1016/j.adhoc.2018.09.004.

13. Whelan J, Sangarapillai T, Minawi O, Almehmadi A, El-Khatib K. Novelty-Based Intrusion Detection of Sensor Attacks on Unmanned Aerial Vehicles. New York, NY, USA: Association for Computing Machinery; 2020.Available from: `https://doi.org/10.1145/3416013.3426446`.

14. Muniraj D, Farhood M. A framework for detection of sensor attacks on small unmanned aircraft systems. In: 2017 International Conference on Unmanned Aircraft Systems (ICUAS); 2017. p. 1189–1198.

15. Kulp P, Mei N. A Framework for Sensing Radio Frequency Spectrum Attacks on Medical Delivery Drones. In: 2020 IEEE International Conference on Systems, Man, and Cybernetics (SMC); 2020. p. 408–413.

16. Ye B, Howells G, Haciosman M. Investigation of Properties of ICmetric in Cloud. In: 2013 Fourth International Conference on Emerging Security Technologies; 2013. p. 107–108.

17. Zhao L, Alipour-Fanid A, Slawski M, Zeng K. Prediction-Time Efficient Classification Using Feature Computational Dependencies. New York, NY, USA: Association for Computing Machinery; 2018.Available from: `https://doi.org/10.1145/3219819.3220117`.

18. Tahir H, Tahir R, McDonald-Maier K. Securing MEMS Based Sensor Nodes in the Internet of Things. In: 2015 Sixth International Conference on Emerging Security Technologies (EST); 2015. p. 44–49.

19. Zhao L., Alipour-F., Slawski M. and Zeng K. Prediction-time Efficient Classification Using Feature Computational Dependencies. In: 2018 Proceedings of the 24th ACM SIGKDD International Conference on Knowledge Discovery London, United Kingdom, ; 2018. p. 2787–2796.

20. H. Tahir, R. Tahir, and K. McDonald-Maier. Securing MEMS Based Sensor Nodes in the Internet of Things. In: 2015 Sixth International Conference Emerg. Secur. Technolgy, 2015, pp. 44–49, 2015.

21. Ali KM, Samawi VW, Al Rababaa MS. The affect of fuzzification on neural networks intrusion detection system. In: 2009 4th IEEE Conference on Industrial Electronics and Applications; 2009. p. 1236–1241.

22. Tan X, Su S, Zuo Z, Guo X, Sun X. Intrusion Detection of UAVs Based on the Deep Belief Network Optimized by PSO. Sensors. 2019;19(24):5529. doi:10.3390/s19245529.

23. Sedjelmaci H, Senouci SM, Messous MA. How to Detect Cyber-Attacks in Unmanned Aerial Vehicles Network? In: 2016 IEEE Global Communications Conference (GLOBECOM); 2016. p. 1–6.

24. Sedjelmaci H, Senouci SM, Ansari N. A Hierarchical Detection and Response System to Enhance Security Against Lethal Cyber-Attacks in UAV Networks. IEEE Transactions on Systems, Man, and Cybernetics: Systems. 2018;48(9):1594–1606. doi:10.1109/TSMC.2017.2681698.

25. Manesh MR, Kenney J, Hu WC, Devabhaktuni VK, Kaabouch N. Detection of GPS Spoofing Attacks on Unmanned Aerial Systems. In: 2019 16th IEEE Annual Consumer Communications Networking Conference (CCNC); 2019. p. 1–6.

Figure_1

Click here to access/download;Figure;Figure 1.png

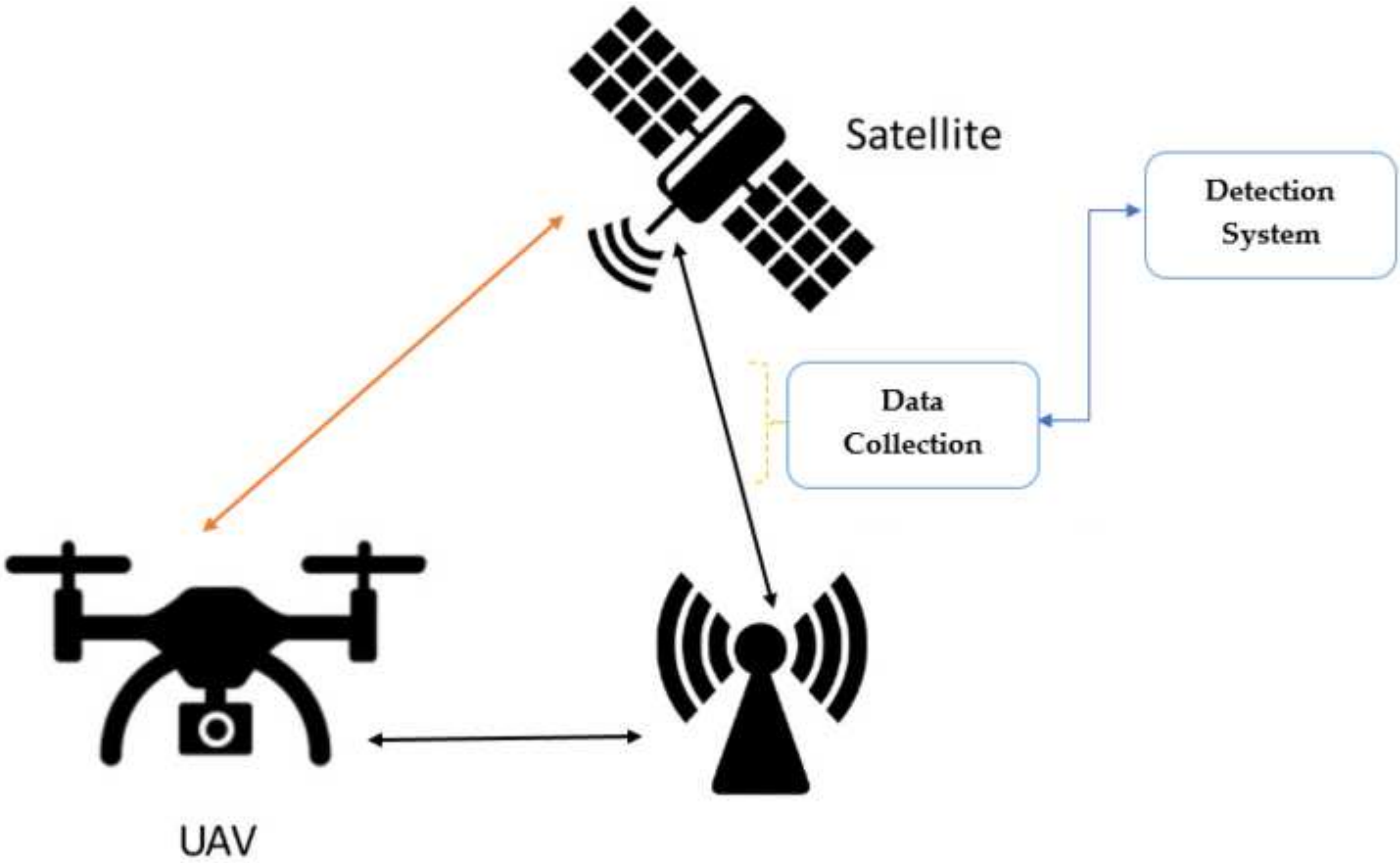

Click here to access/download;Figure;Figure 2.png

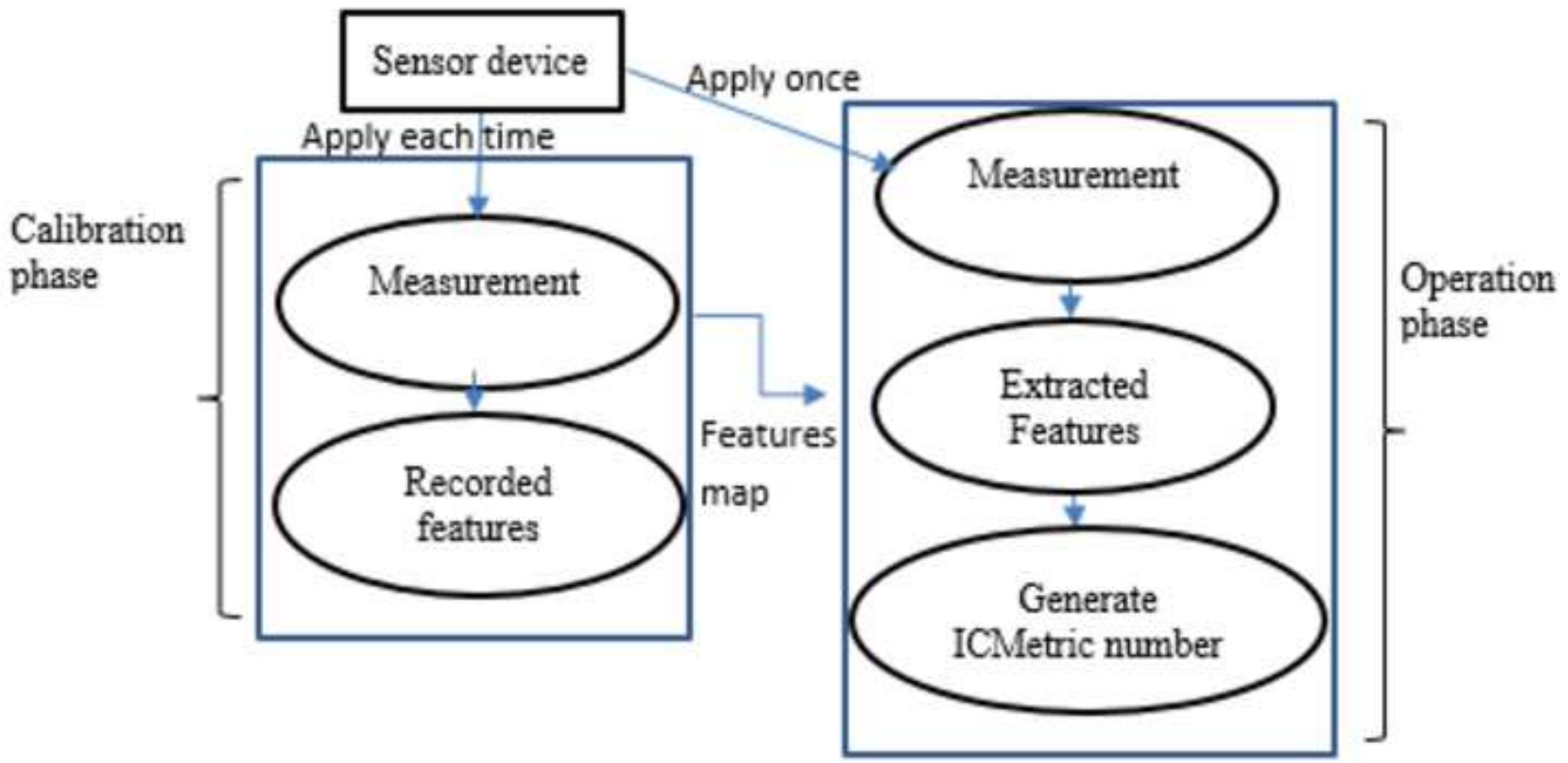

Figure_3

Click here to access/download;Figure;Figure 3.png

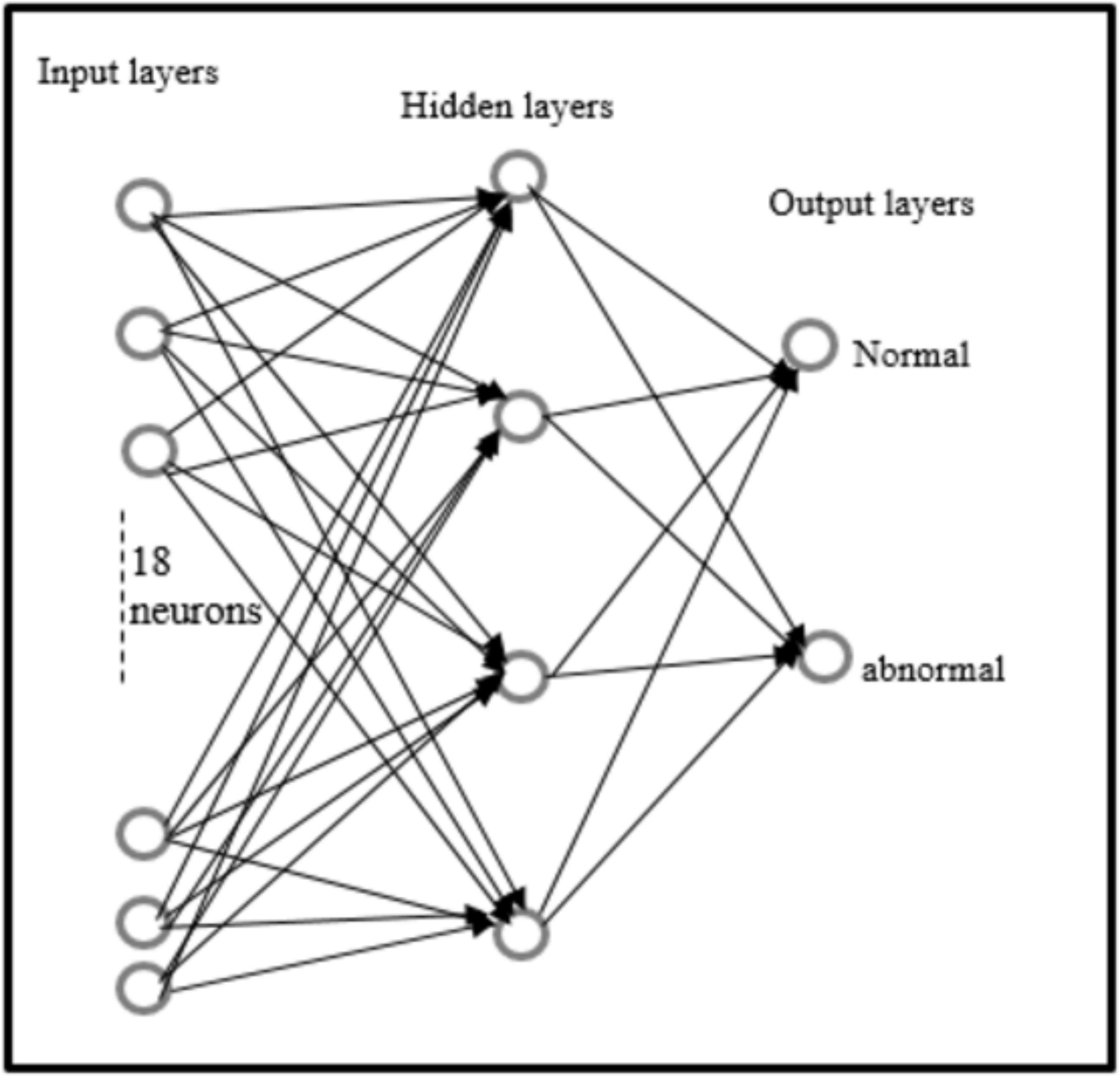

Figure_4 Click here to access/download;Figure;Figure 4.png

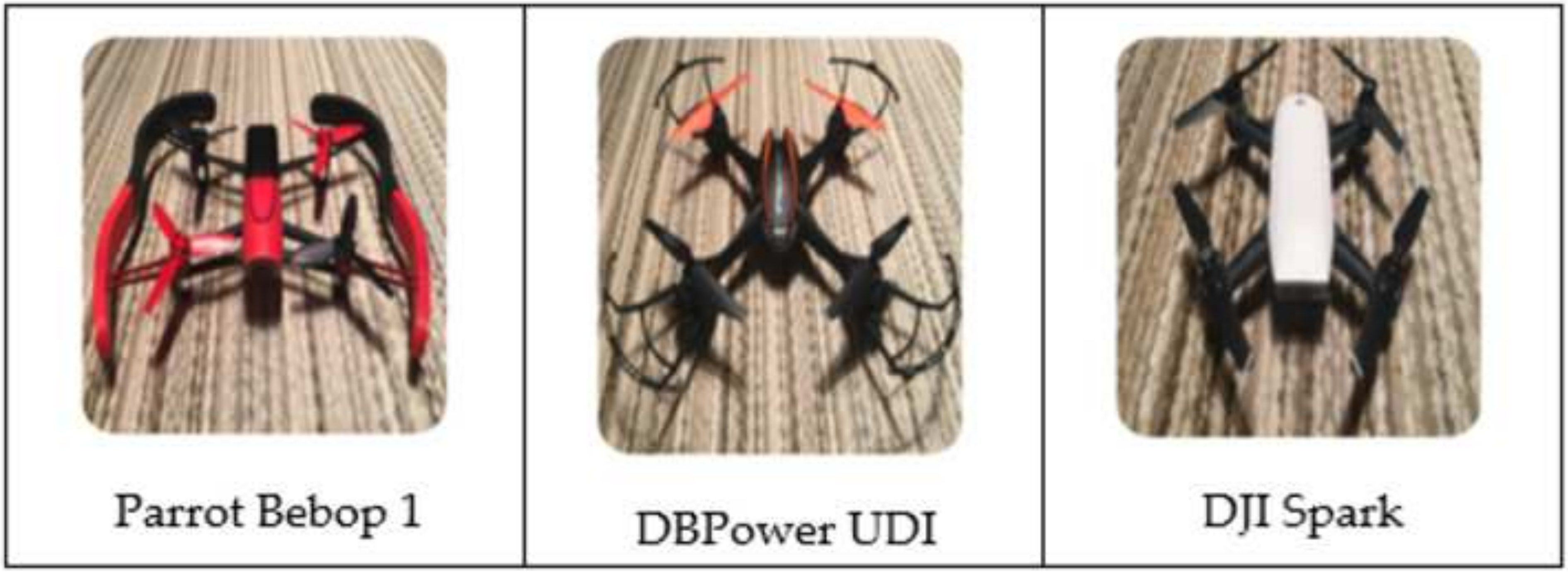

Figure_5

Click here to access/download;Figure;Figure 5.png

Variables - yn

yn

10600x19 double

	1	2	3	4	5	6	7	8	9	10	11	12	13	14
1	3.1307e-06	1.6869e-06	7.3345e-08	7.5223e-07	0.7248	0.4708	1.3182e-06	3.6434e-04	1.6586e-06	0.4318	0.6631	0.6617	0	0.4740
2	2.7158e-06	6.0593e-07	1.8850e-06	2.2943e-05	0.3736	0.0624	1.9435e-07	0	6.2052e-07	0.0141	0.0492	0	0	0.5392
3	6.9806e-07	1.0649e-07	1.4522e-06	3.7235e-05	0.2849	0.0304	2.7882e-08	0	9.0253e-08	0.0248	0.0557	0.0471	0.0207	0.4981
4	4.8264e-06	1.0550e-06	2.1123e-06	1.8430e-05	0.5031	0.2466	7.9267e-07	8.5013e-04	1.1147e-06	0.0113	0.0462	0	0	0.5556
5	9.4113e-07	1.4306e-07	1.8410e-06	1.3352e-05	0.3926	0.0885	5.8716e-08	3.6434e-04	1.3385e-07	0.0141	0.0492	0	0	0.5392
6	2.3482e-06	5.6860e-07	1.9803e-06	3.3662e-05	0.4251	0.1012	2.3519e-07	4.8579e-04	5.7108e-07	0.0141	0.0492	0	0	0.5392
7	7.8460e-07	8.0142e-08	1.9510e-06	1.6925e-05	0.2942	0.0437	2.6160e-08	1.2145e-04	7.7048e-08	0.0141	0.0492	0	0	0.5392
8	7.5453e-07	8.7534e-08	1.9363e-06	3.6107e-05	0.2874	0.0392	2.8538e-08	1.2145e-04	8.0002e-08	0.0174	0.0515	0	0	0.5229
9	2.5460e-06	5.8765e-07	1.5109e-06	3.7611e-05	0.3987	0.0859	2.2511e-07	3.6434e-04	5.9670e-07	0.0250	0.0525	0.0471	0	0.4898
10	4.1472e-06	7.6934e-07	2.1270e-06	2.6140e-05	0.3230	0.0360	2.5807e-07	2.4290e-04	8.3158e-07	0.0109	0.0502	0	0	0.5699
11	1.0677e-06	1.8522e-07	1.9657e-06	3.6107e-05	0.3701	0.0696	6.7491e-08	3.6434e-04	1.7512e-07	0.0202	0.0547	0	0	0.5161
12	2.8967e-06	6.3734e-07	1.7163e-06	4.0244e-05	0.3583	0.0542	2.0575e-07	4.8579e-04	6.5657e-07	0.0212	0.0527	0	0	0.5061
13	7.2765e-07	7.9803e-08	1.6576e-06	3.8740e-05	0.2894	0.0420	2.9030e-08	3.6434e-04	7.2679e-08	0.0141	0.0492	0	0	0.5392
14	7.1871e-07	9.6572e-08	1.7163e-06	4.1749e-05	0.2927	0.0419	4.1741e-08	3.6434e-04	8.4096e-08	0.0160	0.0506	0	0	0.5296
15	1.4385e-06	8.1135e-07	5.8676e-08	3.7611e-07	0.9374	0.8998	8.1629e-07	3.6434e-04	7.7132e-07	0.0383	0.0444	0.0471	0	0.4295
16	2.4619e-06	5.8532e-07	9.6082e-07	2.3507e-05	0.4047	0.0906	2.3913e-07	4.8579e-04	5.9097e-07	0.0259	0.0522	0.0471	0	0.4856
17	1.0734e-06	1.7144e-07	1.9803e-06	3.3662e-05	0.3620	0.0658	6.4621e-08	3.6434e-04	1.6432e-07	0.0151	0.0500	0	0	0.5343
18	1.9401e-06	8.2725e-07	5.8676e-08	3.7611e-07	0.8295	0.7273	7.9390e-07	3.6434e-04	7.9895e-07	0.0410	0.0329	0.0471	0	0.3734
19	4.0927e-06	7.4743e-07	2.3617e-06	2.8020e-05	0.3211	0.0349	2.2158e-07	0.0013	8.0999e-07	0.0099	0.0444	0	0	0.5651
20	1.0246e-06	1.6085e-07	1.9583e-06	3.4414e-05	0.3681	0.0717	5.9700e-08	4.8579e-04	1.5297e-07	0.0141	0.0492	0	0	0.5392

Figure_6 Click here to access/download;Figure;Figure 6.png

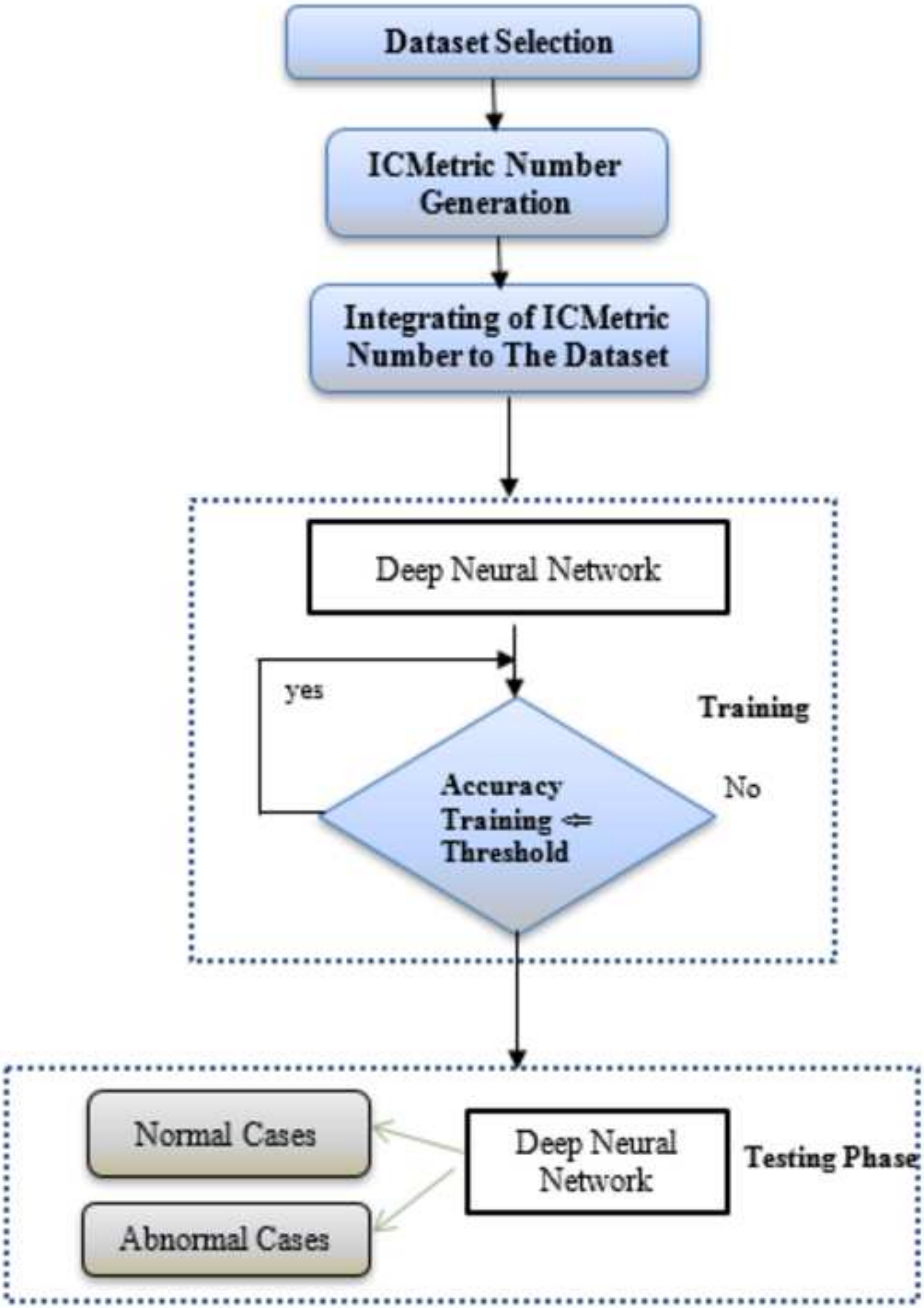

Figure_7

Click here to access/download;Figure;Figure 7.png

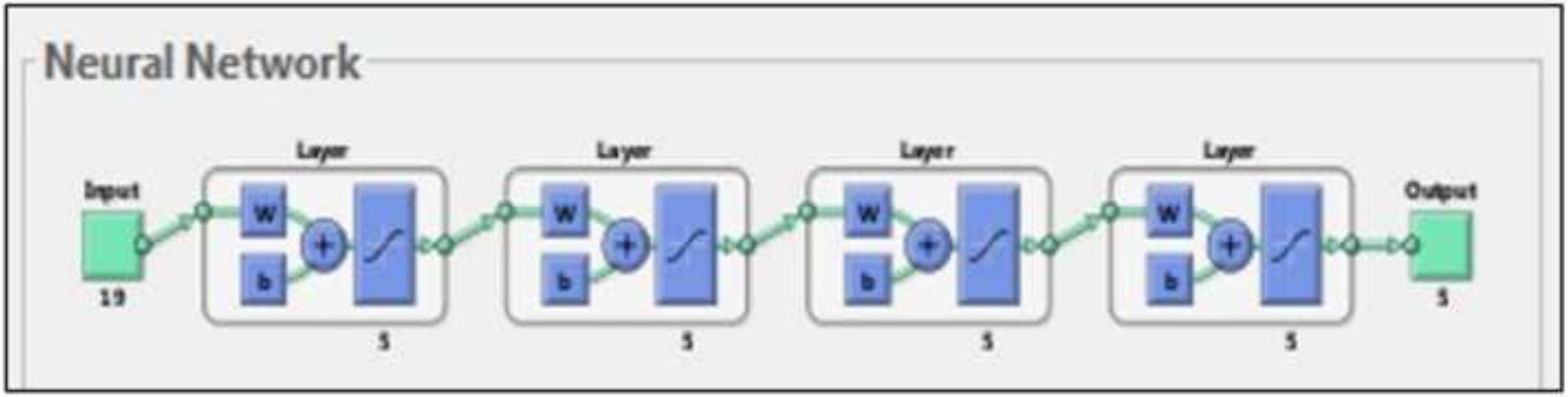

Figure_8

Click here to access/download;Figure;Figure 8.png

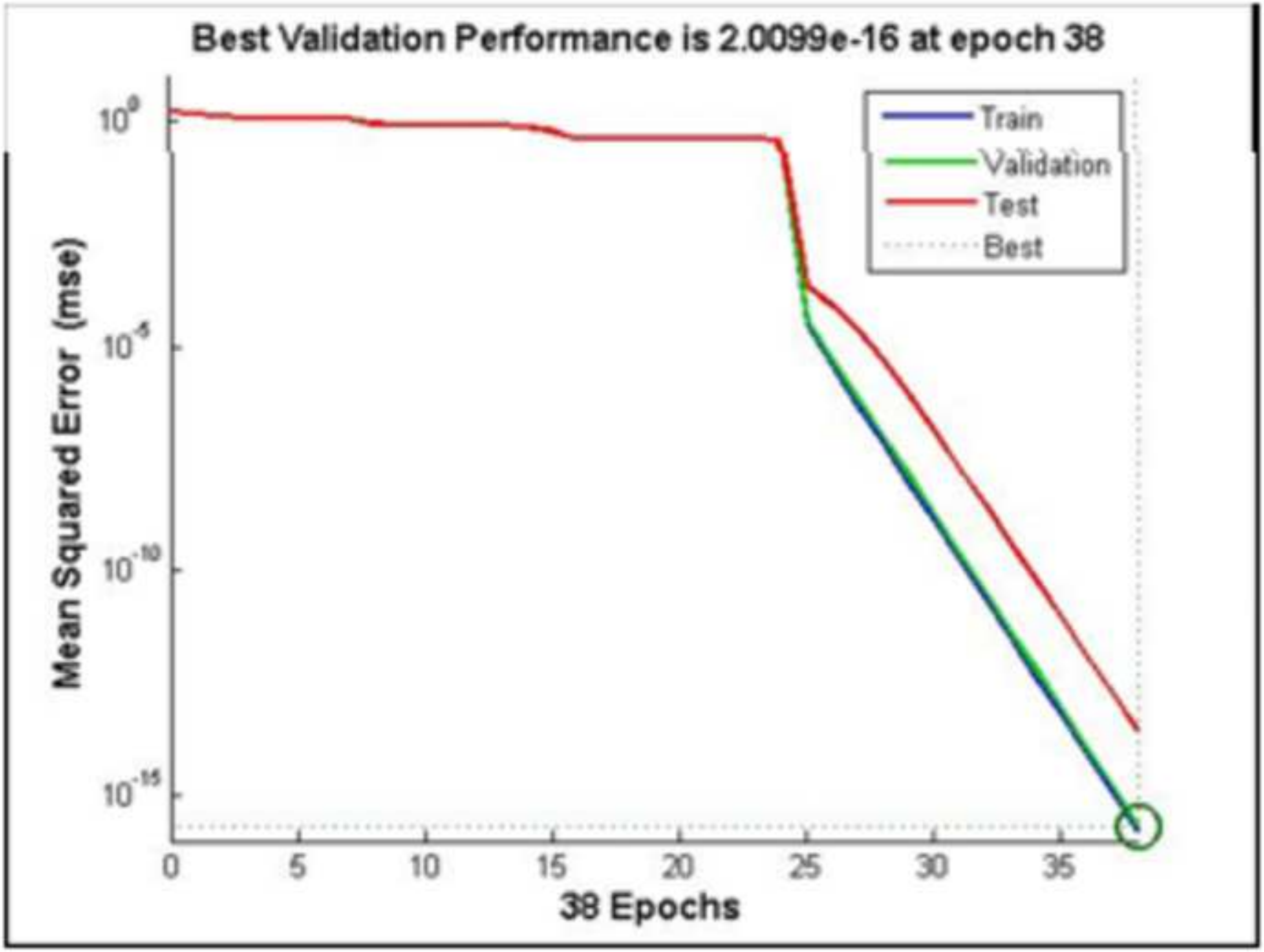

Figure_9

Click here to access/download;Figure;Figure 9.png

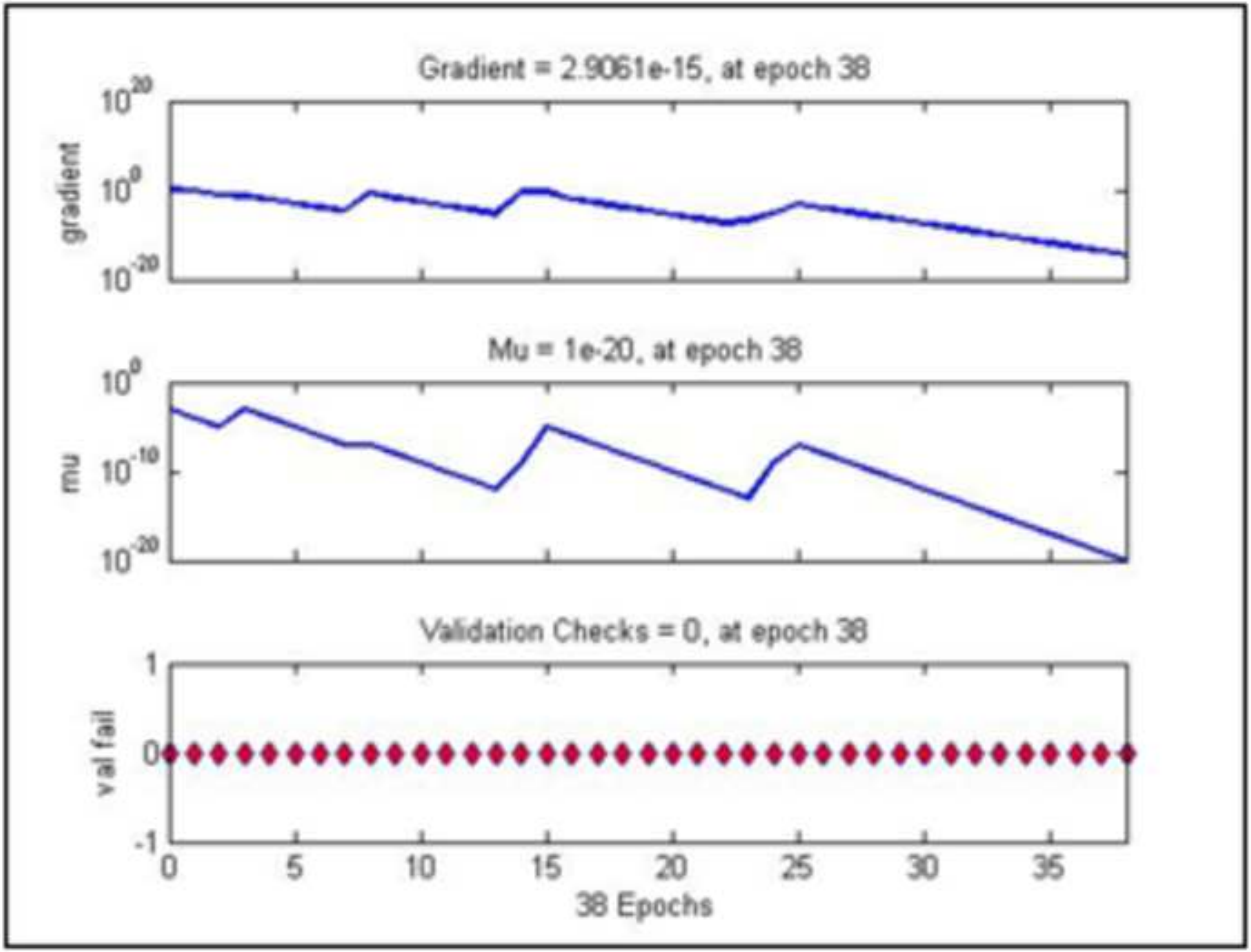

Figure_10

Click here to access/download;Figure;Figure 10.png

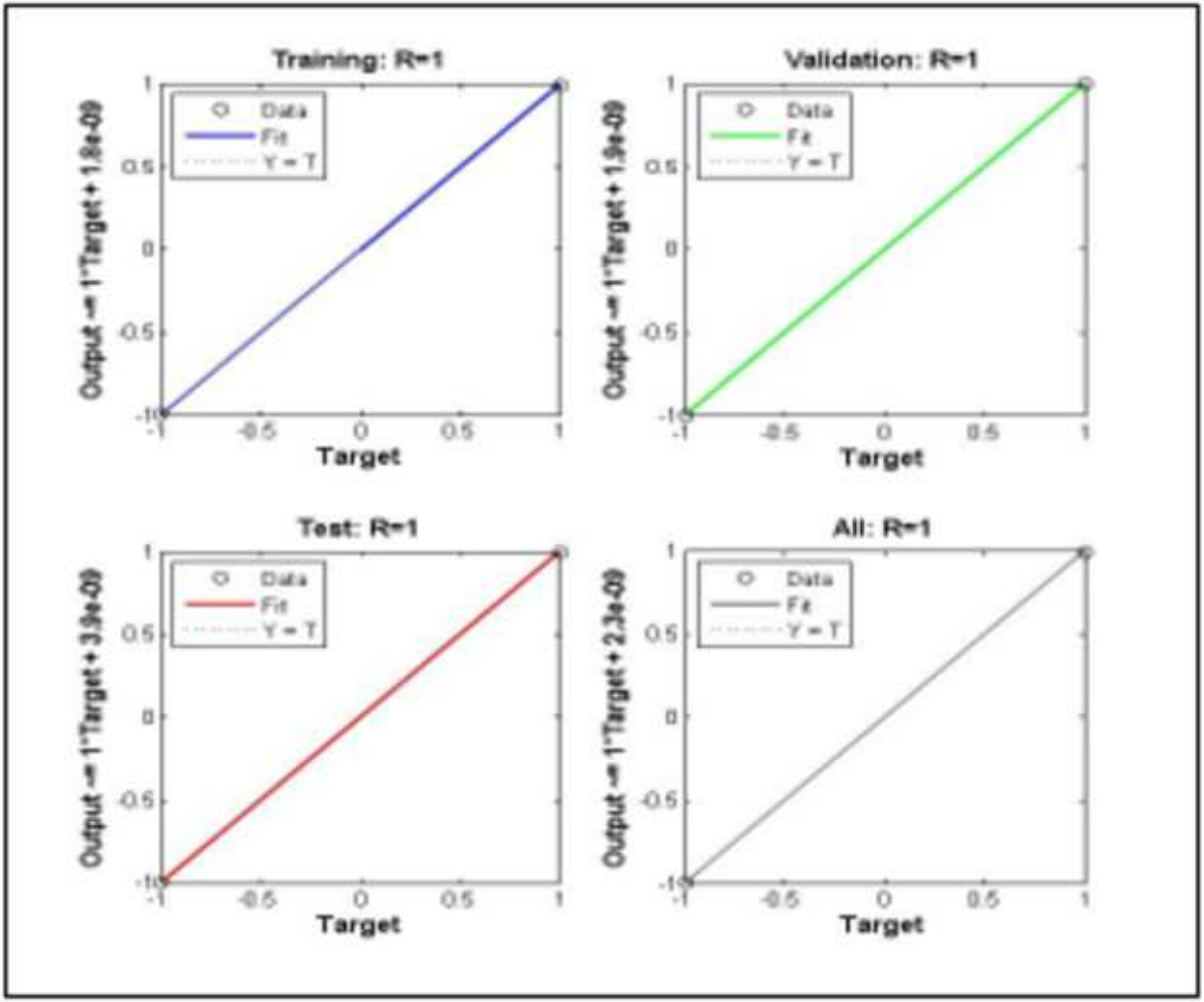

Figure_11

Click here to access/download;Figure;Figure 11.png

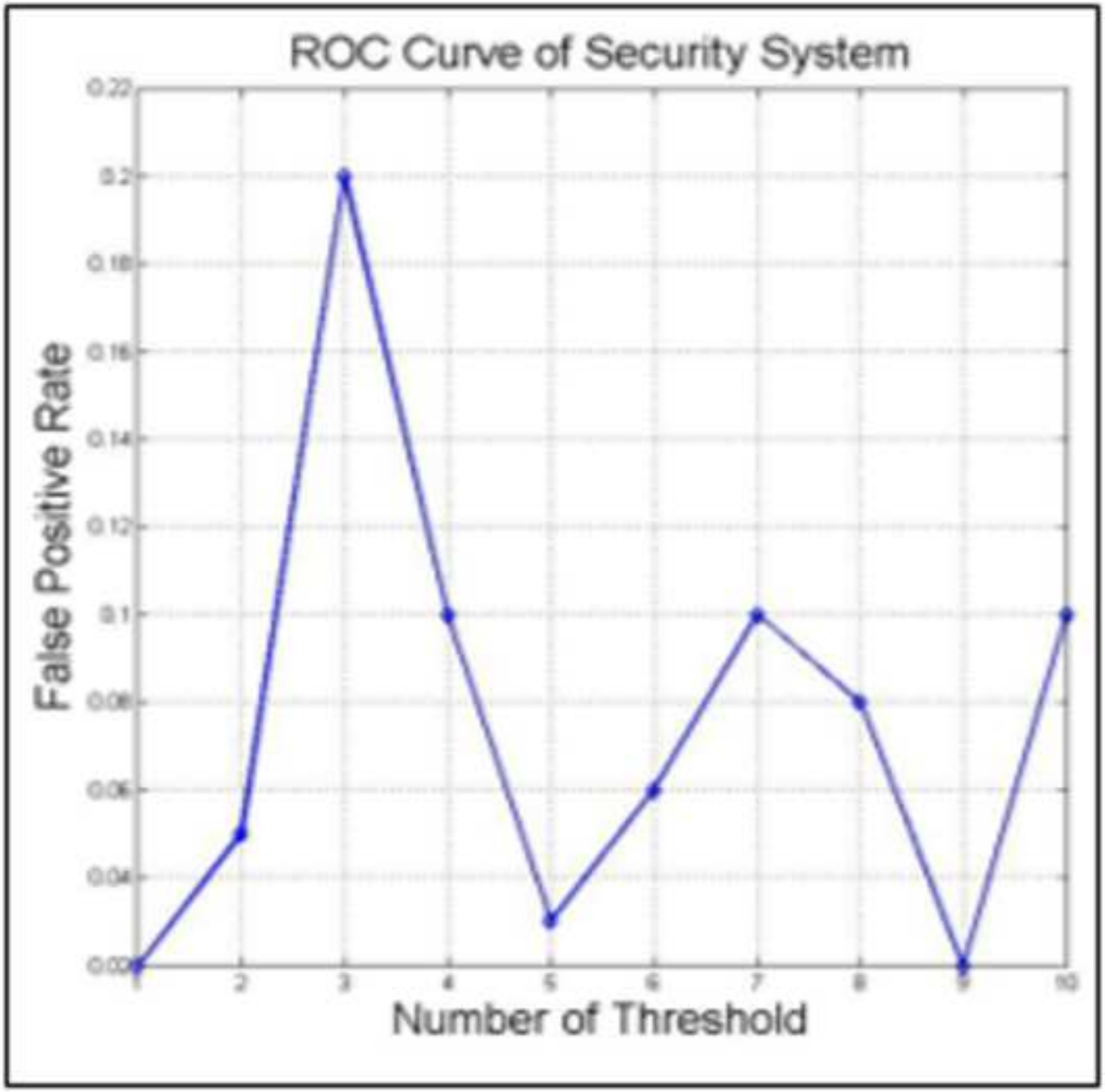

Figure_12 Click here to access/download;Figure;Figure 12.png

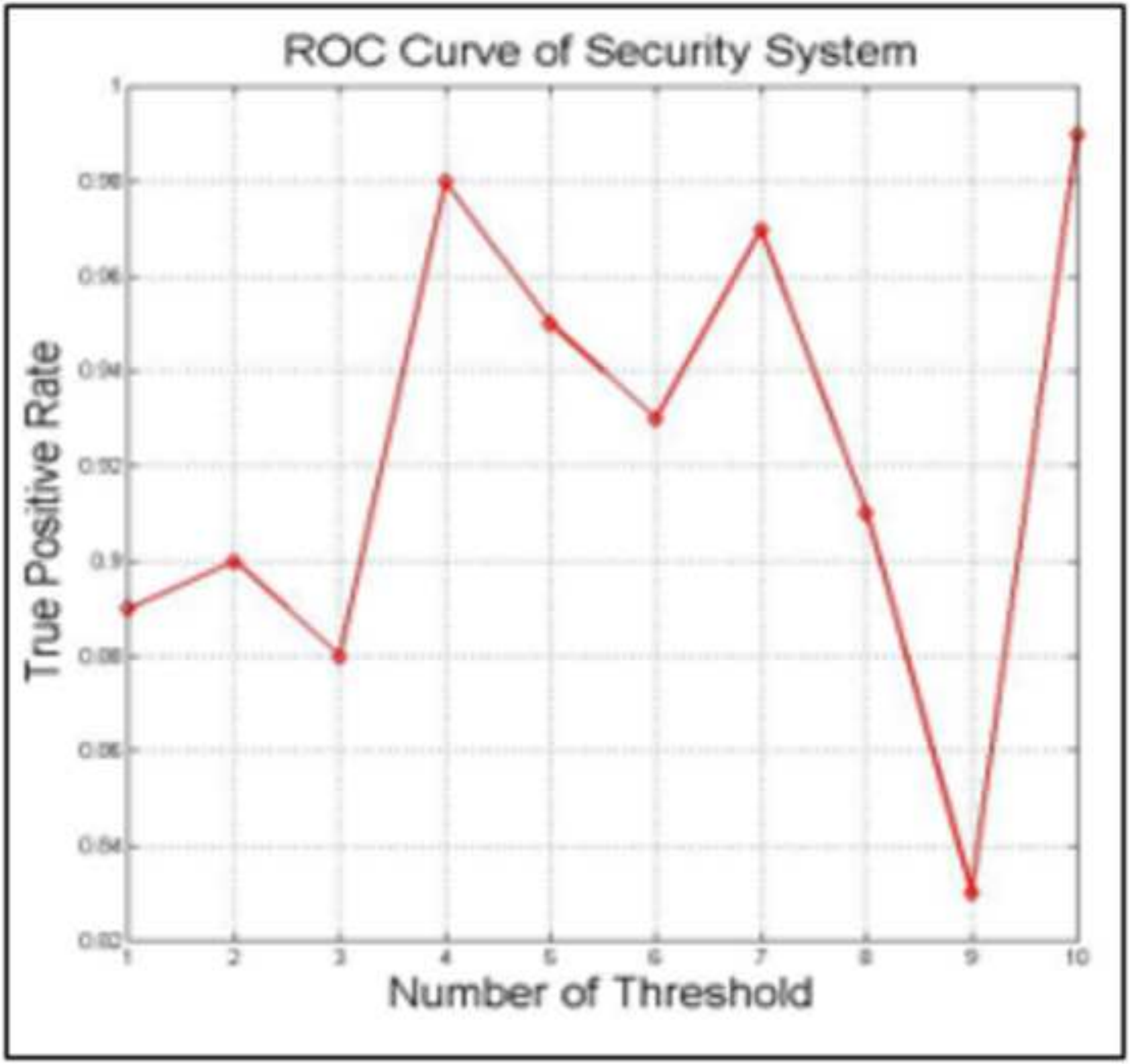

Figure_13 Click here to access/download;Figure;Figure 13.png

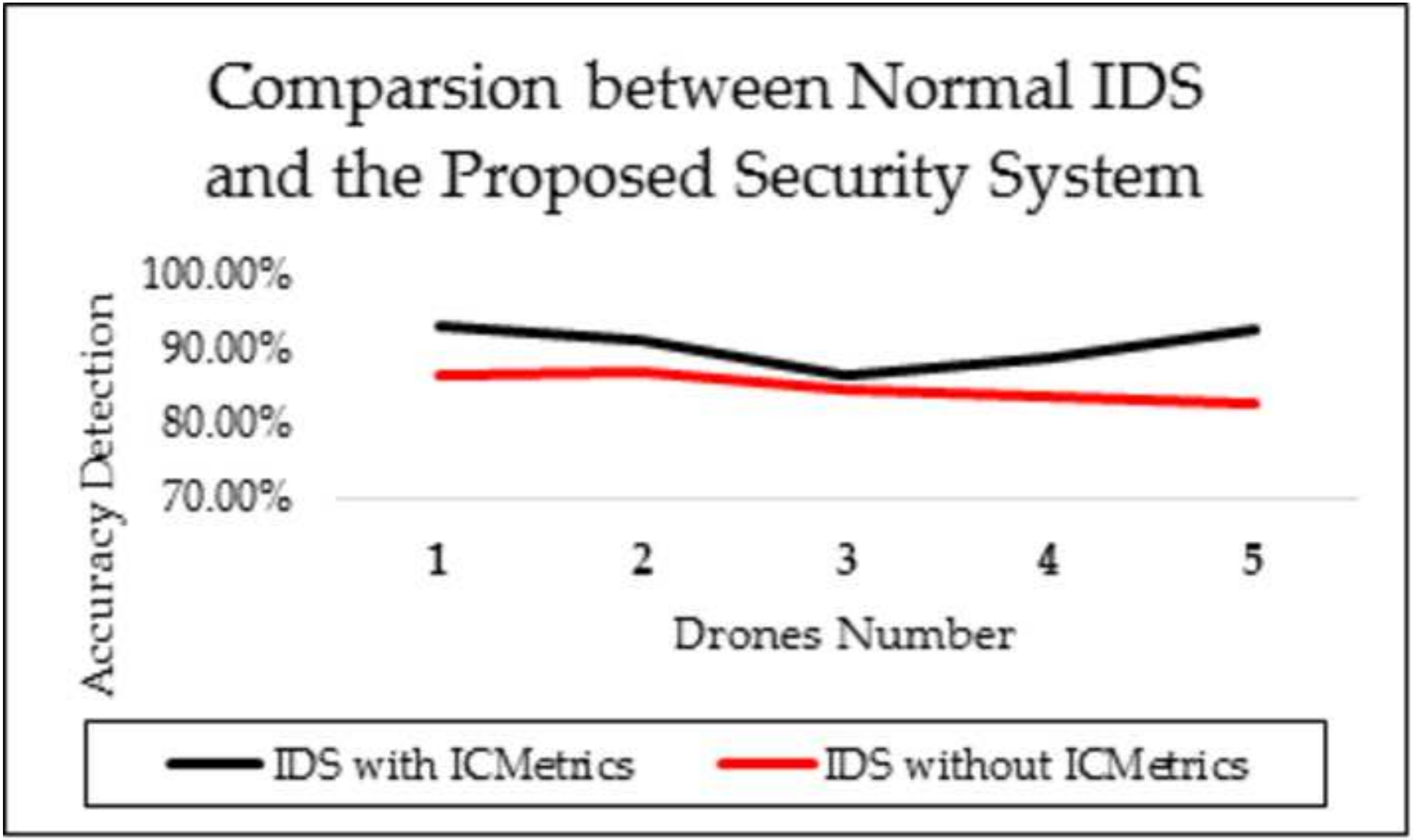

Supplement: Attachment — Submitted filename: PONE-D-21-31928_reviewer.pdf [file pone.0282567.s002.pdf]
