# Supplementary material for: A hybrid security system for drones based on ICMetric technology
Source: PLoS One. 2023 Mar 21;18(3):e0282567. doi: 10.1371/journal.pone.0282567 (PMC10030017; doi:10.1371/journal.pone.0282567)
Supplement: Attachment — Submitted filename: Response to Reviewers.docx [file pone.0282567.s003.docx]

June 27, 2022

**Paper: PONE-D-21-31928, A Hybrid Security System for Drones Based on ICMetric Technology’**

Dear Sir or Madam,

We would like to take this opportunity to thank you for your support and the reviewers for their valuable comments and feedback. We feel that we have made all the changes that were requested and believe that the editor and reviewers will share our opinion that the paper is now much better and suitable for publication.

**Reviewers' Comments**

**Reviewer_1**

**Comment:**

1. The whole article is not well written for a research paper.

**Response**

*We have revised the style of English language as well as all language errors and styles have been proofread.*

2. The format I believe not well maintained.

**Response**

*Done*

3. Some of the sentence seem has no purpose and odd.

**Response**

*Done*

4. I cant really understand the problem of detection as the writer only explained in general about the issue in drone security.

**Response**

*Done*

5. The writer need to polish his writing by addressing the details and sincerely explained the work done.

**Response**

Done

6. I also can’t see any data used in this paper that can help me understand the generation of iCMetric.

**Response**

Figure 5: shows ICMetric is integrated with Wi-Fi traffic data

**Reviewer_2**

**Comment:**

1. The principle of ICMetric has been repeated several times. Similar sentences and messages are observed in Introduction Para, ICMETROC Para, ICMetric Generation and Proposed ICMetric Detection. It may be considered to move all fundamental literature about ICEMetric in one para.

**Response**

*Done*

2. The Proposed ICMetric detection system lacks clarity on the specifics of the system design.

**Response**

*Done*

2. The Proposed DNN lacks clarity on the specifics of the algorithm.

**Response**

*Done*

4. Five neurons have been found to be the most optimized configuration of the hidden layers of the DNN. However, figure 3 shows 4 Neurons.

**Response**

*Done*

5. A Typo in line 162.

**Response**

*Done*

6. It maybe considered to include the details of the proposed system in Table 7 to facilitate an easier comparison.

**Response**

*Done*

7. The Data label of Figure 13 X Axis should be "Number of Drones".

**Response**

*Done*

8. The arrow is intersecting "Apply Each Time" text. The text may be moved to a side.*.*

**Response**

**Done**

Once again, we would like to thank the reviewers and the editorial team for their valuable comments, which have helped us to significantly improve the quality of the paper.

Best Regards,

**Khattab M Ali Alheeti**

University of Anbar
